# Mid-arm muscle area and anthropometry predict low birth weight and poor pregnancy outcomes in Tanzanian women with HIV

**DOI:** 10.1186/s12884-018-2136-z

**Published:** 2018-12-17

**Authors:** Paul Petraro, Isabel Madzorera, Christopher P. Duggan, Donna Spiegelman, Karim Manji, Rodrick Kisenge, Roland Kupka, Wafaie W. Fawzi

**Affiliations:** 1 Department of Nutrition, Harvard TH Chan School of Public Health, 665 Huntington Avenue, Building 1, Room 1102, Boston, MA 02115 USA; 2 Department of Epidemiology, Harvard TH Chan School of Public Health, Boston, MA 02115 USA; 3 Department of Biostatistics, Harvard TH Chan School of Public Health, Boston, MA 02115 USA; 4 Global Health and Population, Harvard TH Chan School of Public Health, Boston, MA 02115 USA; 5 Muhimbili University of Health and Allied Sciences, Dar es Salaam, Tanzania; 6 Division of Gastroenterology and Nutrition, Children’s Hospital Boston, Boston, MA USA; 7 UNICEF Headquarters, New York, NY USA

**Keywords:** Nutrition/wasting, Anthropometry, Muscle area, Women, Pregnancy outcome, Low birth weight, HIV

## Abstract

**Background:**

An observational study was conducted to examine the role of maternal anthropometry, including mid-arm muscle area (MAMA) and others, as risk factors for low birth weight (LBW), small for gestational age (SGA) and preterm births in human immunodeficiency virus (HIV) infected pregnant women. HIV-positive women (*N* = 2369), between 12 and 32 weeks gestation were followed through delivery in Tanzania, from 2003 to 2008. Participants were women enrolled in a randomized, double-blind, placebo-controlled, clinical trial who delivered live births.

**Methods:**

Binomial regression analysis was used to evaluate the association of maternal nutritional indicators of MAMA, mid-upper arm circumference (MUAC), body mass index (BMI) and maternal weight with LBW, SGA and preterm in multivariate analysis.

**Results:**

Higher MAMA was associated with a 32% lower risk of LBW compared to lower measurements (RR = 0.68, 95% CI = 0.50–0.94). Similar protective associations were noted for higher BMI (RR = 0.58, 95% CI = 0.42–0.79); maternal weight (RR = 0.50, 95% CI = 0.36–0.69) and MUAC (RR = 0.62, 95% CI = 0.45–0.86). Higher MAMA was also associated with lower risk of SGA (RR = 0.78, 95% CI = 0.68–0.90) and marginally associated with preterm (RR = 0.85, 95% CI = 0.69–1.04). Beneficial associations of MUAC, BMI and maternal weight with SGA and preterm were also observed.

**Conclusion:**

MAMA performs comparably to MUAC, maternal weight and BMI, as a predictor of LBW and SGA in HIV-infected women. The possible role of MAMA and other indicators in screening HIV positive women at risk of adverse pregnancy outcomes should be investigated.

## Background

Human immunodeficiency virus (HIV) infection, especially advanced disease without retroviral treatment, has been associated with greater risk of adverse pregnancy outcomes, including low birth weight (LBW) and prematurity and infant mortality [1, 2]. Poor nutrition is one of the factors that has been associated with disease progression and poor survival in HIV-infected women [3, 4]. Maternal undernutrition may also be an important contributor to poor pregnancy outcomes in HIV-infected women.

In low income settings, anthropometric measures of body mass index (BMI) and weight gain have been preferred for assessing maternal nutrition status in pregnancy, because they are field friendly, feasible in clinical settings and are widely used [5]. In some settings however, these indicators and others, including maternal height, weight and arm circumference have shown poor sensitivity and specificity in primary screening for women at risk of LBW and SGA births [6, 7]. Thus, there is need for further evaluation of the effectiveness, sensitivity and specificity of these anthropometric tools as indicators of risk of adverse pregnancy outcomes [7], particularly in HIV-infected women.

Previous studies have examined associations between maternal anthropometric measures of nutrition status and pregnancy outcomes, mostly in HIV-negative women [8]. In studies, poor maternal weight gain, BMI and mid-upper arm circumference (MUAC) in pregnancy, have been associated with preterm, SGA and LBW [8–10]. Further, the use of maternal anthropometry indicators of symphysio-fundal height and373 abdominal girth as surrogate measures of birth weight have been piloted in Tanzania [11]. Low BMI, poor weight gain during pregnancy and anemia have also been linked to adverse pregnancy outcomes in HIV-positive women [4, 5, 12–18].

The development and testing of novel anthropometric tools, such as mid-arm muscle area (MAMA) in resource poor settings is important. MAMA is a reliable measure of muscle atrophy and thickness of subcutaneous fat in lean patients [19]. MAMA may therefore be valuable in evaluating nutrition status for HIV positive women, given that muscle wasting is a marker of HIV disease progression [4]. A recent study in Malawi found that women with higher MAMA had lower odds of having LBW infants [20]. Studies have also shown that MUAC, which is strongly associated with MAMA [21], is associated with body weight, malnutrition and increased risk of death in post-partum women [21–23].

Studies have not extensively evaluated the performance of MAMA as a predictor of adverse pregnancy outcomes. Further, the performance of MAMA in relation to other anthropometric indicators, in predicting poor pregnancy outcomes for HIV-positive women has not been elucidated. In a prospective study, we assess the performance of MAMA as an alternative to traditional maternal anthropometry indicators in predicting poor pregnancy outcomes in a Tanzanian sub-population.

## Methods

### Study design and setting

The aim of the study was to examine the role of maternal anthropometry as a risk factor for LBW, SGA and preterm births in HIV-positive pregnant women. The parent study was a randomized, double-blind, placebo-controlled clinical trial of high dose multivitamins (vitamin B complex, C and E) compared with Recommended Dietary Allowance (RDA) for HIV-infected women in Dar es Salaam, Tanzania. Study participants were women aged ≥18 years, who presented for antenatal care at ≤32 weeks of pregnancy at 1 clinic between August 2004 and November 2007 in Dar es Salaam. Participants intended to stay in Dar es Salaam for 2 years. Details of the study have been published elsewhere [24]. Infants born to the HIV-positive women were enrolled in a trial to evaluate whether micronutrient supplementation reduces risk of death and other adverse outcomes [24]. Inclusion criteria for the child study were singleton (no twins or triplets), live births of infants of HIV-positive women. Exclusion criteria were serious congenital anomalies and medical conditions for children. This analysis includes all eligible women with children that were randomized into the child study, who completed a questionnaire at baseline and completed antenatal visits prior to delivery. Written informed consent was obtained from the women at study enrolment.

### Study procedures

At enrollment trained research personnel administered questionnaires to collect socio-demographic data for women and collected baseline measurements of gestational age, maternal weight and height. Other baseline information collected included medical history and clinical examination with blood collection. Trained medical personnel also conducted assessment of CD4 counts at initial and subsequent follow-up visits. Maternal weight and height were measured on repeat visits. Maternal height was measured to the nearest 0.1 cm and weight was measured to the nearest 100 g. MUAC was measured on the left arm at the midpoint between the olecranon and the acromion, using a non-stretchable tape. Measurements were to the nearest 0.1 cm. Detailed information about the infant was obtained at delivery. This included birth weight, head circumference, height, and general health. HIV-1 sero-status in women was assessed using 2 sequential ELISAs that used Murex HIV antigen/antibody (Abbot Murex) followed by the Enzygnost anti–HIV-1/2 Plus (Dade Behring). Discordant Elisa test results were resolved using a Western blot assay.

Standard of care for HIV treatment was offered to women in accordance to the Tanzania National Guidelines. At study inception routine care included malaria prophylaxis, iron and folate supplementation, and treatment of sexually transmitted infections and opportunistic infections. All women were given Nevirapine prophylaxis for mother to child transmission at birth (one dose to the mother at labor onset and one dose to infant within 72 h of birth) [25, 26]. By 2005, standard care included anti-retroviral treatment for women with WHO classified stage IV HIV disease or CD4 cell count of 200 cells/μL, or WHO stage III and CD4 cell count of 350 cells/μL [24]. The women were followed throughout the study and continued to receive care and treatment in the program including multivitamin supplementation. Women who were started on antiretroviral therapy (ART) however were changed to single RDA multivitamin dosages.

### Ascertainment of risk factors

The exposures of interest were MAMA, MUAC, BMI and maternal weight gain during pregnancy. BMI was evaluated as weight in kilograms divided by square of height in meters. MAMA was calculated using the mid-upper arm circumference and trifold skin measurements taken during antenatal visits.

The formula used to calculate MAMA was as follows:

(((MUAC (cm) – pi *(trifold skin measurement (mm)/10)**2)-6.5) / pi *4) [27].

MAMA calculations are prone to overestimation caused by assuming a circular mid-arm muscle compartment and the inclusion of mid-arm cross-sectional bone area. MAMA calculations were corrected for bone content by subtracting a mid-arm bone area of 6.5cm^2^ as specified for women in Heymsfield [27]. Similar assessments for mid-arm muscle mass have been used in other studies [25, 28].

### Study outcomes

The primary study outcomes were LBW, SGA and preterm birth. LBW was defined as weight under 2500 g at delivery, SGA was defined as birth weight less than the 10th percentile for gestational age, according to standards of Oken [29], and preterm birth was defined as birth prior to 37 weeks gestation. Gestational age was established based on maternal menstruation history.

### Ethics

The Research and Publications Committee of Muhimbili University of Health and Allied Sciences and the Institutional Review Board of the Harvard T.H. Chan School of Public Health approved the study protocol.

### Statistical analysis

We modeled the effects of MAMA, BMI, MUAC and maternal weight gain on pregnancy outcomes of LBW, SGA and preterm birth in a cohort of 2369 HIV-positive women with live born children. Univariate and multivariate models using log binomial regression were used to estimate the relative risk (RR) between risk factors and outcomes [30]. The RR was selected instead of odds ratios for easier interpretability and given that the odds ratio may overestimate the RR for common outcomes [30].

We defined mean MAMA and MUAC as the average measure for each woman from all prenatal visits that we had data on. The majority of women had at least 2 antenatal visits, with the average number of visits per woman being 4.8 (median = 5 visits). The slopes of the curves were calculated as the change in MAMA, maternal weight, MUAC and BMI for each woman. Given that changes in measures over gestation could be a source of bias, we examined the effect of overall changes in maternal weight, MAMA, MUAC and BMI and additionally, changes in these measures in the 2nd trimester on LBW, SGA and preterm, because major development of the fetus occurs during this period. We attempted to assess measures in the 3rd trimester, but due to limited repeated measures there was insufficient power to assess significance (less than 10% of women had multiple visits in the 3rd trimester, excluding delivery). The mean change for each woman was computed and women were then ranked, with those in the lowest tercile having the lowest change in anthropometric measure and those in the highest tercile having the greatest change during the period of follow up.

The Wald test statistic was used to assess significance of associations between risk factors and outcomes. Exposures of MAMA, BMI, MUAC and maternal weight during pregnancy were evaluated as continuous variables. For change in anthropometric measures during pregnancy (BMI, MUAC, maternal weight), a slope was modeled for each participant and tertiles used to categorize the variables.

We considered confounding by baseline risk factors. We controlled for known confounders of maternal age (mean < 28 years/≥28 years), gestational age (mean < 25 /≥25 weeks) or gestational age at enrollment, WHO clinical stage of HIV (1,2,3,4), family member with diabetes (yes/no), previous LBW baby (yes/no), previous caesarian delivery (yes/no), previous baby died in first 7 days (yes/no), malaria during pregnancy determined using positive blood smear (yes/no), repeated fever during this pregnancy (yes/no), and hypertension during this pregnancy (defined by standards used by Kilewo [31], yes/no). Additional confounders considered included marital status (married/ other), education (none, 1–4, 5–8, 8+ years), employment (none, informal income, formal income), previous pregnancies (0, 1 to 3, 3+) and daily food expenditure (< 500 Tsh/> 500 Tsh). We identified potential confounders based on univariate association with exposure variables with a *p*-value less than 0.20. We believe this is a conservative approach and will account for unmeasured confounding.

Conventional cut-offs were used to categorize risk factors where available; otherwise medians were used to classify the variables that were not normally distributed. Risk factors were also examined continuously and we explored non-linearity of the relationships using stepwise restricted cubic splines [32]. We examined risk factors in both ways to assess their potential association with LBW, SGA, and preterm birth. In a sensitivity analysis we restricted the analysis to 1015 HIV positive women who were in WHO HIV stage 1, which was defined as asymptomatic persistent generalized lymphadenopathy (PGL) (or CD4 count > 500 cells/μL).

The final model included all significant variables from the univariate based criteria and selected confounders. Observations with missing data were retained in the analyses by using missing indicators. We assumed that missingness is conditionally independent of the outcome and thus unbiased [33].

All analyses were done using SAS, Version 9.1, statistical software from SAS Institute Inc. (Cary, NC, USA).

## Results

**Fig. 1:**
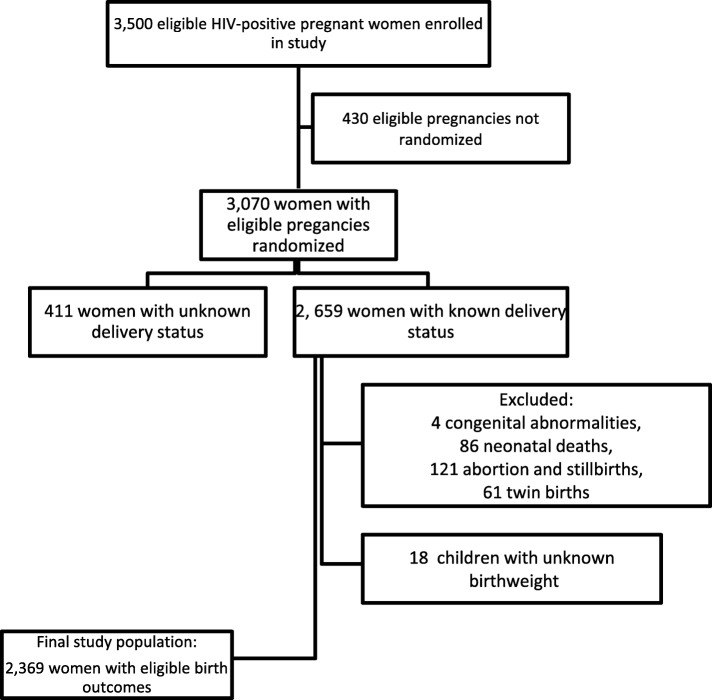
Flow chart for selection of study population

The sample size of the parent study was 3500 HIV-positive women selected to evaluate whether micronutrient supplementation could reduce the risk of mortality and infectious disease morbidity. The analysis sample was composed of 2369 women with available birth outcomes. The flow diagram for the study is shown in Fig. 1 below.

Table 1 shows the key baseline characteristics of the study population. The mean age of women in the study population was 28.3 years (±5) and on average women were enrolled in the study at the gestational age of 24.6 weeks (±5.5). The mean weight for women at enrollment was 59.1 kg (±10.9) and mean MUAC was 26.1 cm (±3.2). Of the women assessed, 22.9% (*N* = 522) were primiparous, 86.9% (*N* = 1977) were married or living with a partner, and 80% (*N* = 1803) had less than 8 years of education. The majority of the infants in the study were born at full term, with mean gestational age at delivery of 39.4 weeks (±2.5) and mean birth weight for infants was 3.1 kg (±0.5) (not shown in results).

**Table 1:** Baseline Characteristics

Maternal characteristics	N (%)
Age (years)	28.3 (5)^a^
Gestational age at enrollment (weeks)	24.6 (5.5)^a^
Marital status	
Married/living with partner	1977 (86.9)
Single	298 (13.1)
Previous pregnancies	
0 (primiparous)	522 (22.9)
1 to 3	1574 (69.1)
3 +	182 (7.9)
Education	
None	151 (6.6)
1–4 years	85 (3.7)
5–8 years	1567 (68.8)
8 + years	476 (20.9)
Employment	
None (housewife)	1474 (64.1)
Informal income (housewife with income)	463 (20.1)
Formal income	279 (12.1)
Daily food expenditure (per person/day/Tsh^b^)	
< 500	1117 (51.7)
≥ 500	1044 (48.3)
WHO stage (HIV)	
1	1015 (72.6)
2	214 (15.3)
3	125 (8.9)
4	45 (3.2)
Obstetric history	N (%)
Previous low birth weight baby	128 (5.6)
Previous preterm baby	115 (5)
Previous caesarian delivery	100 (4.4)
Previous baby died within first few days	405 (17.6)
Previous stillbirth	94 (4.1)
Previous abortion (less than 7 months)	423 (18.4)
Family member with diabetes	230 (10)
Current pregnancy characteristics	N (%)
Malaria (prior to enrollment)	724 (31.5)
Repeated fever (prior to enrollment)	224 (9.7)
Malaria prophylaxis (prior to enrollment)	722 (31.4)
Baseline CD4 count (enrollment) (cells/ml)	264.8 (219.1)^a^
Baseline weight (at enrollment) (kg)	59.1 (10.9)^a^
Mid Upper Arm Circumference (at enrollment)(cm)	26.1 (3.2)^a^
Mid-Arm Muscle Area (at enrollment)(cm)	32.6 (7.4)^a^
Mean systolic blood pressure (at enrollment) (mm/hg)	108.8 (13.2)^a^
Mean diastolic blood pressure (at enrollment) (mm/hg)	65.5 (9.2)^a^
Adherence to iron supplements (fraction adhered during pregnancy)	0.65 (0.4)^a^
Average prenatal visits	7.8 (3.0)^a^

^a^Mean and standard deviation. Means presented are based on baseline measures only;^b^SVD Standard vaginal delivery; TShs, Tanzanian shillings (US dollar is estimated at approximately 1200 shillings)

Of the deliveries with a record for birth weight, 7% (*N* = 161) were LBW babies (less than 2500 g), with 1.1% (*N* = 25) being very low birth weight babies (VLBW, less than 2000 g). 25.5% (*n* = 587) of the live births were SGA (below the 10th percentile), and 15.6% (*n* = 359) were preterm infants.

### Low birth weight

Table 2 shows the results of binomial regression analysis of the associations of MAMA, BMI, MUAC and maternal weight gain in pregnancy with LBW. HIV positive mothers with mean MAMA > 33.1 cm had 32% lower risk of LBW (RR = 0.68, 95% CI = 0.50–0.94) compared to women with MAMA of 33.1 cm or lower in adjusted models. Women with mean MUAC > 26 cm also had 38% lower risk of LBW (RR = 0.62, 95% CI = 0.45–0.86) compared to women with lower MUAC. Further, having a BMI > 25.7 (mean for women) in pregnancy was associated with a 42% lower risk of LBW (RR = 0.58, 95% CI = 0.42–0.79) and women with mean weight greater than 61.8 kg had a 50% lower risk of LBW (RR = 0.50, 95% CI = 0.36–0.69). Complete results of univariate and multivariate analysis can be found in Table 2.

**Table 2:** Anthropometric Risk Factors for Low Birth Weight

Characteristics	N	Univariate		Multivariate^a^	
		RR (95% CI)	*P*-Value	RR (95% CI)	*P*-Value
Maternal weight change - overall (slope)					
Tertile 1	47	Reference	0.003^	Reference	0.29^
Tertile 2	30	0.62 (0.40, 0.97)		0.62 (0.40, 0.96)	
Tertile 3	25	0.53 (0.33, 0.85)		0.61 (0.38, 0.98)	
Maternal weight change - 2nd trimester (slope)					
Tertile 1	38	Reference	0.01^&^	Reference	0.60^
Tertile 2	26	0.67 (0.41, 1.08)		0.66 (0.41, 1.06)	
Tertile 3	22	0.58 (0.35, 0.97)		0.67 (0.40, 1.12)	
BMI change - overall (slope)					
Tertile 1	48	Reference	0.001^&^	Reference	0.11^&^
Tertile 2	34	0.69 (0.45, 1.05)		0.66 (0.44, 1.01)	
Tertile 3	21	0.44 (0.27, 0.72)		0.45 (0.27, 0.74)	
MAMA mean (cm)					
≤ 33.1	91	Reference		Reference	
> 33.1	60	0.70 (0.51, 0.96)	0.0002^	0.68 (0.50, 0.94)	0.009^
Mama change - overall (slope)					
Tertile 1	45	Reference	0.85^		
Tertile 2	48	1.04 (0.70, 1.54)			
Tertile 3	42	0.93 (0.62, 1.40)			
MAMA change - 2nd trimester (slope)					
Tertile 1	38	Reference	0.87^		
Tertile 2	33	0.84 (0.53, 1.32)			
Tertile 3	37	0.97 (0.63, 1.50)			
MUAC mean (cm)					
≤ 26 cm	99	Reference		Reference	
> 26 cm	52	0.61 (0.44, 0.84)	0.0001^	0.62 (0.45, 0.86)	0.0006^
MUAC change - overall (slope)					
Tertile 1	36	Reference	0.64^		
Tertile 2	27	0.73 (0.45, 1.19)			
Tertile 3	34	0.94 (0.59, 1.47)			
MUAC change - 2nd trimester (slope)					
Tertile 1	30	Reference	0.66^		
Tertile 2	23	0.75 (0.44, 1.27)			
Tertile 3	30	0.98 (0.60, 1.60)			
Adherence to iron supplementation					
< Median	80	Reference		Reference	
≥ Median	77	0.85 (0.63, 1.14)	0.03^	0.94 (0.69, 1.28)	0.34^

^ Test for Trend

^&^ Association was significantly non-linear. *P*-value corresponds to test for overall significance

^a^Multivariate Model adjusted for all variables in the univariate with a *p*-value less than 0.20 plus maternal age (< 28, ≥ 28 years); gestational age (< 25, ≥ 25 weeks);WHO stage (1,2,3,4); family member with diabetes, previous low birth weight baby, previous caesarian delivery, previous baby died with first 7 days, malaria during pregnancy, repeated fever during this pregnancy, and hypertension during this pregnancy. Anthropometric variables (weight, BMI, MAMA) were entered into separate multivariate models. Where both mean and change (slope) variables are significant they were both entered into the same model (e.g. weight and weight change)

### Small for gestational age

In Table 3 we show associations of MAMA, BMI, MUAC and maternal weight gain with SGA. Higher MAMA (> 33.1 cm) was associated with 22% lower risk of SGA (RR = 0.78, 95% CI = 0.68–0.90). The risk of SGA was lower in women with BMI > 25.7 during pregnancy (RR = 0.69, 95% CI = 0.59, 0.79), and in women with MUAC> 26 cm (RR = 0.75, 95% CI = 0.65 = 0.87). Other significant risk factors for SGA in the multivariate models included mean weight > 61.8 kg (RR = 0.63, 95% CI = 0.54–0.73) and increase in weight during pregnancy comparing women in second tertile (RR = 0.75, 95% CI = 0.63–0.89) and third tertile (RR = 0.58, 95% CI = 0.47–0.71) to those in the first tertile.

**Table 3:** Anthropometric Risk Factors for Small for Gestational Age

Characteristics	N	Univariate		Multivariate^a^	
		RR (95% CI)	*P*-Value	RR (95% CI)	*P*-Value
Maternal weight change - overall (slope)					
Tertile 1	203	Reference	< 0.0001^	Reference	< 0.0001^
Tertile 2	159	0.76 (0.64, 0.91)		0.75 (0.63, 0.89)	
Tertile 3	107	0.53 (0.43, 0.65)		0.58 (0.47, 0.71)	
Maternal weight change - 2nd trimester (slope)			< 0.0001^		
Tertile 1	172	Reference		Reference	< 0.0001^
Tertile 2	129	0.73 (0.61, 0.88)		0.72 (0.60, 0.87)	
Tertile 3	89	0.52 (0.42, 0.65)		0.56 (0.45, 0.70)	
BMI change - overall (slope)					
Tertile 1	201	Reference	< 0.0001^	Reference	< 0.0001^
Tertile 2	156	0.75 (0.63, 0.89)		0.75 (0.63, 0.88)	
Tertile 3	113	0.56 (0.46, 0.69)		0.59 (0.49, 0.72)	
MAMA mean (cm)					
≤ 33.1	330	Reference		Reference	
> 33.1	239	0.76 (0.66, 0.88)	< 0.0001^	0.78 (0.68, 0.90)	< 0.06^
MAMA change - overall (slope)					
Tertile 1	193	Reference	0.69^		
Tertile 2	183	0.94 (0.79, 1.12)			
Tertile 3	171	0.89 (0.74, 1.06)			
MAMA change - 2nd trimester (slope)					
Tertile 1	162	Reference	0.19^	Reference	0.63^
Tertile 2	167	1.00 (0.83, 1.70)		0.99 (0.83, 1.19)	
Tertile 3	135	0.84 (0.69, 1.02)		0.84 (0.70, 1.03)	
MUAC (mean)					
≤ 26 cm	347	Reference		Reference	
> 26 cm	222	0.74 (0.64, 0.85)	< 0.0001^	0.75 (0.65, 0.87)	< 0.0001^
MUAC change - overall (slope)					
Tertile 1	148	Reference	0.99^		
Tertile 2	155	1.02 (0.85, 1.24)			
Tertile 3	133	0.90 (0.73, 1.10)			
MUAC change - 2nd trimester (Slope)					
Tertile 1	118	Reference	0.72^		
Tertile 2	128	1.06 (0.86, 1.31)			
Tertile 3	116	0.97 (0.78, 1.20)			
Adherence to iron supplementation					
< median	272	Reference		Reference	
≥ median	315	0.85 (0.63, 1.14)	0.01^	1.08 (0.93, 1.24)	0.28^

^ Test for Trend

^a^ Multivariate Model adjusted for all variables in the univariate with a *p*-value less than 0.20 and marital status (married, other), education (none, 1–4 years, 5–8 years, 8+ years), employment (none, informal income, formal income), previous pregnancies (0, 1 to 3, 3+), daily food expenditure (< 500 Tsh, > 500 Tsh);WHO stage (1,2,3,4), previous low birth weight baby, previous caesarian delivery, previous baby died with first 7 days, malaria during pregnancy, and hypertension during this pregnancy. Anthropometric variables (weight, BMI, MAMA) were entered into separate multivariate models. Where both mean and change (slope) variables are significant they were both entered into the same model (e.g. weight and weight change

Additionally, women with greater increase in weight during second trimester of pregnancy [second tertile vs. first (RR = 0.72, 95% CI = 0.60 = 0.87) and third tertile vs. first (RR = 0.56, 95% CI = 0.45–0.70)] had lower risk of SGA births. Finally, greater change in BMI during pregnancy was protective against SGA for those in the second tertile (RR = 0.75, 95% CI = 0.63–0.88) and third tertile (RR = 0.59, 95% CI = 0.49–0.72) compared to those in the lowest tertile. There a statistically significant non-linear relationship between mean BMI and SGA (results not shown).

### Preterm birth

Table 4 presents results of the association between maternal nutritional status and preterm birth. MAMA greater than 33.1 cm was marginally associated with preterm births in the multivariate analysis (RR = 0.85, 95% CI = 0.69–1.04). Higher maternal BMI (> 25.7) was associated with a 27% lower risk of preterm (RR = 0.73, 95% CI = 0.59–0.89) and having a higher mean weight (> 61.8 kg) was associated with a 25% lower risk of preterm (RR = 0.75, 95% CI = 0.61–0.92). Women with higher MUAC (> 26 cm) had 30% lower risk of preterm (RR = 0.70, 95% CI = 0.56–0.86). There was a statistically significant non-linear relationship between MAMA and preterm births (results not shown).

**Table 4:** Anthropometric Risk Factors for Preterm birth

Characteristics	N	Univariate		Multivariate^a^	
		RR (95% CI)	*P*-Value	RR (95% CI)	*P*-Value
Maternal weight change - overall (slope)					
Tertile 1	56	Reference	0.68^		
Tertile 2	61	1.06 (0.75, 1.50)			
Tertile 3	67	1.20 (0.86, 1.68)			
Maternal weight change - 2nd trimester(slope)					
Tertile 1	47	Reference	0.32^		
Tertile 2	60	1.24 (0.87, 1.78)			
Tertile 3	63	1.34 (0.94, 1.92)			
BMI change - overall (slope)					
Tertile 1	58	Reference	0.21^	Reference	0.14^
Tertile 2	59	0.99 (0.70, 1.39)		0.99 (0.70, 1.39)	
Tertile 3	67	1.16 (0.83, 1.61)		1.16 (0.83, 1.61)	
MAMA (mean) (cm)					
≤ 33.1	167	Reference		Reference	
> 33.1	134	0.84 (0.68, 1.04)	0.07^	0.85 (0.69, 1.04)	0.04^&^
MAMA change - overall (slope)					
Tertile 1	76	Reference	0.93^		
Tertile 2	113	1.45 (1.10, 1.90)			
Tertile 3	73	0.96 (0.71, 1.30)			
MAMA change - 2nd trimester (slope)					
Tertile 1	68	Reference	0.61^		
Tertile 2	48	0.68 (0.48, 0.97)			
Tertile 3	68	1.00 (0.73, 1.37)			
MUAC (mean) (cm)					
< 26 cm	189	Reference		Reference	
≥ 26 cm	112	0.68 (0.55, 0.85)	0.002^	0.70 (0.56, 0.86)	0.002^
MUAC change - overall (slope)					
Tertile 1	71	Reference	0.82^		
Tertile 2	37	0.51 (0.35, 0.74)			
Tertile 3	64	0.89 (0.65, 1.23)			
MUAC change - 2nd trimester (slope)					
Tertile 1	67	Reference	0.86^		
Tertile 2	34	0.50 (0.34, 0.73)			
Tertile 3	61	0.90 (0.65, 1.23)			
Adherence to iron supplementation					
< median	219	Reference		Reference	
≥ median	140	0.64 (0.52, 0.78)	< 0.0001&	0.72 (0.59, 0.89)	< 0.0001^&^

^ Test for trend

^&^ Association was significantly non-linear. P-value corresponds to test for overall significance

^a^ Multivariate Model adjusted for all variables in the univariate with a p-value less than 0.20 plus gestational age (< 25, ≥ 25 weeks); education (none, 1–4, 5–8, 8+ years), previous pregnancies (0, 1 to 3, 3+), daily food expenditure (< 500 Tsh, > 500 Tsh); previous low birth weight baby, previous baby died with first 7 days, previous stillbirth, previous abortion (less than 7 months), repeated fever during pregnancy. Anthropometric variables (weight, BMI, MAMA) were entered into separate multivariate models. Where both mean and change (slope) variables are significant they were both entered into the same model (e.g. weight and weight change)

## Discussion

We evaluated the associations of MAMA, MUAC, BMI, maternal weight and pregnancy weight gain with adverse pregnancy outcomes in HIV-infected women in Tanzania. We found that maternal anthropometric indicators consistently showed a protective effect against adverse pregnancy outcomes. Improved anthropometric measures were associated with lower risk of LBW, SGA and preterm, although MAMA was only marginally associated with preterm birth. Thus, MAMA performed comparably to other maternal anthropometric measures in predicting poor pregnancy outcomes. These results are consistent with findings from a previous study that poor maternal nutrition status may be a risk factor for adverse pregnancy outcomes in HIV-positive populations [20].

Our findings further support the use of maternal anthropometry as a tool for assessing nutrition status and screening for adverse pregnancy outcomes including LBW, SGA, and preterm in the developing world, given low costs, resource requirements and ease of implementation. Simpler tools such as attained weight at 16–20 or 24–28 weeks of pregnancy and change in maternal weight during pregnancy may be more practical screening instruments for LBW and intrauterine growth restriction (IUGR) in primary care settings [34]. In addition, maternal BMI during pregnancy also has predictive properties. For example, given that changes in maternal anthropometric measures over gestation, as well as the timing of measurements may influence pregnancy outcomes, we evaluated associations of changes in MAMA, maternal weight, MUAC and BMI throughout pregnancy (overall slope of the curve) and second trimester slopes (MAMA, maternal weight, MUAC) with pregnancy outcomes. Greater overall changes in maternal weight and BMI and changes in weight in the 2nd trimester were protective against SGA. Similar associations has been found elsewhere [35].

However, these tools have limitations. For women with below average pre-pregnancy weight, the greatest effect size for IUGR prediction is for attained weight at 7 lunar months [34], which may not allow sufficient time for intervention on fetal growth before birth [36]. Further, in low income settings equipment for height measurements may not be available, thus the calculation of BMI may not be convenient. It is thus important to consider tools such as MUAC (in second or third trimesters as it is strongly associated with infant mortality in the first week of life [37]) or the proposed MAMA tool.

MAMA was highly correlated with and performed comparatively to established maternal nutrition indicators of weight gain, BMI and MUAC as a predictor of adverse outcomes. Our findings also provide construct validation of MAMA as a measure maternal nutrition status in our location. Thus, MAMA could also be useful as a measure of nutritional status for HIV positive women during pregnancy in low resource settings. While MAMA involves complex calculations, its key advantage over other indicators is its ability to measure muscle mass in lean patients [19]. This attribute makes MAMA a suitable tool for measurements of wasting in HIV-infected women and for evaluating progression of HIV disease. HIV disease is characterized by increased protein catabolism and decreased muscle mass, and lower muscle mass may be an important risk factor for mortality in people with HIV [38]. Further, MAMA has been associated with mortality in chronic diseases such as chronic obstructive pulmonary disease and in hemodialysis patients [39, 40]. The association of MAMA with mortality could be an even more important consideration given high risk for mortality in HIV-positive women.

Similarly, MUAC could also be a useful tool. MUAC is similarly associated with adverse pregnancy outcomes [41], even in HIV-positive populations [20], yet it is not routinely measured in primary care settings. Further, conducting MUAC measurements may be easier than assessing skinfold thickness and calculating MAMA. Follow-up research is required to further evaluate both the use of MAMA and MUAC indicators, as well as comparing sensitivity and specificity of various cut off points for these indicators in HIV positive women.

Previous studies have found mixed results of associations between maternal weight, arm circumference, abdominal circumference, BMI, and weight gain and LBW and SGA in HIV negative populations [42, 43]. Our findings suggest greater utility for maternal anthropometry in HIV positive populations, where the tracking and measuring of maternal nutrition status is of paramount importance for both maternal health and the health of the offspring.

HIV infection in Tanzania remain high, with prevalence of nearly 6% among women of child bearing age [44]. Although the advent of universal HIV treatment and adoption of option B+ treatment, that is the provision of lifelong antiretroviral (ARV) treatment to all pregnant and breastfeeding HIV-positive women (regardless of CD4 count or clinical stage) [45] may have contributed to improved nutrition status, the coverage of programs including prevention of mother-to-child transmission (PMTCT) of HIV has not reached many women [46]. In our study sample the prevalence of ARV drug use was 20.2%. Coverage of antiretroviral therapy in Tanzanian women has since then increased to 78% [47], and ARV use has been associated with weight gain and improved anthropometric status [48]. However, given that knowledge of HIV status remains at 70% [47], we still concerned about maternal underweight during pregnancy and its effects on pregnancy outcomes among untreated, HIV-infected women. Further, in a study in Tanzania, HIV-positive women, the majority of whom were on ART experienced a greater risk of adverse outcomes (fetal death, preterm delivery and LBW) [49]. Thus, while our study may be dated, our findings may still be relevant in the period of option B+ and other HIV treatment regimes.

This may also be true in other contexts. In a Ugandan study, HIV-positive women on combination antiretroviral therapy with low gestational weight gain had increased occurrence of LBW, preterm births, and other poor birth outcomes [50]. Approximately 15% of the women experienced weight loss during pregnancy and 44.9% were anemic [50]. Finally, protein-calorie malnutrition and food insecurity remain barriers in improving nutrition status and may still hasten HIV disease progression [51]. This suggests that malnutrition is still a problem for women on ART and that it can have consequences for child health, hence the need for addressing maternal undernutrition in pregnancy in this population.

Finally, with the advent of treatment, there are additional considerations that need to be studied to fully understand the impact of treatment on this population. This can be evaluated with anthropometric indicators. The initiation of option B+ programs provides an opportunity to integrate MAMA and other anthropometric tools into routine care and treatment of women with HIV. Given evidence in this study and others that MAMA, MUAC, BMI and weight change in pregnancy are predictive of adverse pregnancy outcomes, these tools can be used to identify women at greater risk of adverse outcomes. Secondly, anthropometric indicators may be useful for identifying women to be targeted by nutrition interventions. For these purposes, anthropometric indicators such as MAMA must be further evaluated.

A limitation of our study was that it did not further evaluate the sensitivity and specificity of MAMA and other anthropometric indicators in the study population. This research question can potentially be addressed in future studies now that we have demonstrated a possible role for MAMA and other indicators in this population. Research is further required to evaluate whether MAMA and other evaluated anthropometric indicators could be feasibly used as screening tools for pregnant women with HIV in different locations and environments. Further, there were fewer repeated anthropometric measures in the third trimester of pregnancy. Thus, we could not evaluate associations between anthropometric measures in the third trimester and adverse outcomes. As a result, the study may have excluded important associations for which the third trimester is most important. Finally, we evaluated gestational age using maternal menstrual history and were not able to verify gestational age with the ultrasound measurements which are more accurate, given their high costs and unavailability in low resource areas [52]. Menstrual history was used as in many limited resource settings, because it is easy and inexpensive to collect, however it is prone to measurement error due to poor maternal recall [53]. Other neonatal data methods e.g. Dubowitz and Ballard scoring systems for estimation of child gestational age may also be accurate, however they may be less reliable in malnourished populations and require technical skills which may not be available [54]. Our study design though observational, is prospective in nature. A major strength of this study was the ability to assess various anthropometric measures simultaneously. To our knowledge this is the first study to compare the performance of MAMA with standard measures of anthropometry in a large sample of HIV-infected women.

## Conclusion

MAMA performs comparably to MUAC, maternal weight and BMI as a predictor of LBW and SGA in HIV-infected women. The possible role of MAMA and other indicators in screening HIV-positive women at risk of adverse outcomes should be investigated.

## Abbreviations

ART: Antiretroviral therapy
ARV: Antiretroviral
BMI: Body mass index
HIV: Human immunodeficiency virus
IUGR: Intrauterine growth restriction
LBW: Low birth weight
MAMA: Mid-arm muscle area
MUAC: Mid upper arm circumference
PGL: Persistent generalized lymphadenopathy
PMTCT: Prevention of mother-to-child transmission
RDA: Recommended Dietary Allowance
RR: Relative risk
SGA: Small for gestational age
VLBW: Very low birth weight babies
WHO: World Health Organization
